# The effects of kiwifruit consumption on anthropometric and cardiometabolic indices in adults: A systematic review and meta‐analysis

**DOI:** 10.1002/fsn3.4385

**Published:** 2024-08-09

**Authors:** Pedram Pam, Mohammad Ali Goudarzi, Shirin Ghotboddin Mohammadi, Omid Asbaghi, Ladan Aghakhani, Cain C. T. Clark, Mohammad Hashem Hashempur, Neda Haghighat

**Affiliations:** ^1^ Student Research Committee, Department of Clinical Nutrition Tabriz University of Medical Sciences Tabriz Iran; ^2^ Department of Clinical Nutrition Tabriz University of Medical Sciences Tabriz Iran; ^3^ Shahrekord Branch Islamic Azad University Shahrekord Iran; ^4^ Department of Clinical Nutrition, School of Nutrition and Food Sciences Isfahan University of Medical Sciences Isfahan Iran; ^5^ Cancer Research Center Shahid Beheshti University of Medical Sciences Tehran Iran; ^6^ Student Research Committee Shahid Beheshti University of Medical Sciences Tehran Iran; ^7^ Laparoscopy Research Center Shiraz University of Medical Sciences Shiraz Iran; ^8^ College of Life Sciences Birmingham City University Birmingham UK; ^9^ Research Center for Traditional Medicine and History of Medicine, Department of Persian Medicine, School of Medicine Shiraz University of Medical Sciences Shiraz Iran

**Keywords:** anthropometric indices, cardiometabolic indices, integrative medicine, kiwifruit, lipid profile, systematic review, traditional Persian medicine

## Abstract

The current systematic review and meta‐analysis was conducted to evaluate the effects of kiwifruit intake on anthropometric indices and key cardiometabolic parameters. Related articles were found by searching PubMed, ISI Web of Science, and Scopus to detect relevant Randomized Clinical Trials (RCTs) and novel systematic reviews relating to kiwi consumption in adults, up to August 2023. The weighted mean difference (WMD) and 95% confidence intervals (CIs) were calculated using a random‐effects model. Heterogeneity, sensitivity analysis, and publication bias were assessed and reported using standard methods. Six RCTs were included in the meta‐analysis. Analyzing overall effect sizes demonstrated a significant reduction in low‐density lipoprotein cholesterol (LDL) levels (WMD: −9.30 mg/dL; 95% CI: −17.56 to −1.04, *p* = .027), whereas no significant alterations of triglycerides (TG) (WMD: −12.91 mg/dL; 95% CI: −28.17 to 2.34, *p* = .097), total cholesterol (TC) (WMD: −7.66 mg/dL; 95% CI: −17.85 to 2.52, *p* = .141), high‐density lipoprotein cholesterol (HDL) (WMD: 2.87 mg/dL; 95% CI: −0.36 to 6.11, *p* = .141), fasting blood glucose (FBG) (WMD: 1.06 mg/dL; 95% CI: −1.43 to 3.56, *p* = .404), C‐reactive protein (CRP) (WMD: 0.15 mg/dL; 95% CI: −0.40, 0.70, *p* = .0598), body weight (BW) (WMD: 0.85 kg; 95% CI: −1.34 to 3.04, *p* = .448), body mass index (BMI) (WMD: 0.04 kg/m^2^; 95% CI: −0.75 to 0.83, *p* = .920), and waist circumference (WC) (WMD: 0.18 cm; 95% CI: −1.81 to 2.19, *p* = .855) were found. Our findings suggest that consuming kiwifruit does not have a significant impact on anthropometric indices and cardiometabolic factors, except for LDL‐C levels.

## Graphical abstract

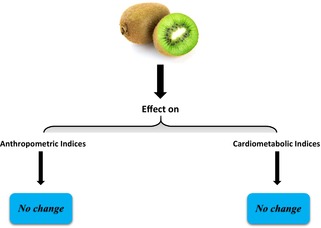

## 1 INTRODUCTION

The global burden of cardiometabolic diseases, such as obesity, diabetes, and cardiovascular disorders, continues to rise, posing a significant and sustained public health challenge. These conditions not only contribute to increased morbidity and mortality rates but also place a substantial economic burden on healthcare systems worldwide (Artime et al., 2021; Ferdinand, 2018; Gao et al., 2021; Miranda et al., 2019; Stol et al., 2021; Vaduganathan et al., 2022). The prevalence of obesity and type 2 diabetes has reached epidemic proportions, now representing a profound impact on individuals' quality of life and overall well‐being (Ampofo & Boateng, 2020; Jaacks et al., 2019; Khan et al., 2020; Sun et al., 2022; Tinajero & Malik, 2021; Wolfenden et al., 2019). Furthermore, cardiovascular diseases remain the leading cause of death globally (Jagannathan et al., 2019; Mc Namara et al., 2019; McClellan et al., 2019), and as such, addressing the complex interplay of factors contributing to these conditions, including genetic, environmental, and lifestyle influences, is imperative for mitigating the escalating burden of cardiometabolic disorders (Kokubo et al., 2019; Münzel et al., 2020, 2022; Zhang et al., 2021).

Indeed, identifying dietary interventions and nutritional approaches that may positively influence cardiometabolic health has become a focal point in public health research and clinical practice. The recognition of the profound impact of diet on the development and progression of cardiometabolic diseases has underscored the importance of exploring dietary patterns, specific food components, and nutritional strategies as potential tools for disease prevention and management (Brauer et al., 2021; Darani et al., 2023; Hedayati et al., 2023; Jardon et al., 2022; Remde et al., 2022; Sharifi‐Rad et al., 2020).

In light of the escalating burden of cardiometabolic disorders, there is an increasing emphasis on developing evidence‐based dietary recommendations and interventions that can effectively mitigate risk factors and improve outcomes for individuals at risk of, or already affected by, these conditions. This shift toward a more comprehensive understanding of the role of nutrition in cardiometabolic health has significant implications for public health policies, clinical guidelines, and individualized patient care, highlighting the need for continued research and innovation in the field of nutritional science (Aghabeiglooei et al., 2023; Belardo et al., 2022; Casula et al., 2022; Ghoreishi et al., 2023; Goudarzi et al., 2023; Kordafshari et al., 2015; Seidu et al., 2023; Zarshenas et al., 2016).

Kiwifruit (*Actinidia deliciosa*) has garnered attention in recent years due to its potential health benefits. Rich in essential nutrients, including vitamins C and K, dietary fiber, and phytochemicals, kiwifruit has emerged as a promising dietary component with diverse physiological effects (Satpal et al., 2021; Singletary, 2012; Stonehouse et al., 2013; Zehra et al., 2020; Zhuang et al., 2019). The unique nutritional composition of kiwifruit, coupled with its antioxidant and anti‐inflammatory properties, has sparked scientific interest in exploring its potential role in promoting overall health and preventing chronic diseases. Additionally, the presence of bioactive compounds, such as polyphenols and flavonoids, in kiwifruit has been putatively associated with various health‐promoting effects, including improved cardiovascular function, enhanced immune response, and potential modulation of metabolic pathways (D'Eliseo et al., 2019; Maheshwari et al., 2022; Mishra, Ishfaq, et al., 2022; Saeed et al., 2019; Suksomboon et al., 2019; Zhang et al., 2020; Zuraini et al., 2021).

Consuming 2–3 kiwifruit daily, which provide 280–420 mg of vitamin C, for 28 weeks has been shown to lower platelet aggregation and circulating triglyceride levels (Mishra, Bentley‐Hewitt, et al., 2022). Monro et al. (2022) demonstrated that consuming two kiwifruits with breakfast resulted in an increased intake of antioxidant nutrients, without affecting fasting insulin levels. In addition, a cross‐sectional study with 1469 participants discovered that consuming at least one kiwi per week is linked to decreased levels of fibrinogen and triglycerides in the blood, as well as increased levels of HDL‐cholesterol (Recio‐Rodriguez et al., 2015). Another randomized controlled trial study showed that in men and women with moderately elevated blood pressure (BP), 24‐h systolic and diastolic BP were lower following the consumption of three kiwifruits daily for 8 weeks (Svendsen et al., 2015).

As a result, investigating the impact of kiwifruit consumption on cardiometabolic indices has become a subject of considerable research, with a growing body of evidence suggesting its potential as a functional food for supporting cardiometabolic health in adults. The findings from various studies have contributed to a growing body of evidence that informs our understanding of the potential health benefits associated with incorporating kiwifruit into the adult diet (Becerra‐Tomás et al., 2021; Duttaroy & Jørgensen, 2004; Khalua et al., 2020; Mishra, Bentley‐Hewitt, et al., 2022; Recio‐Rodriguez et al., 2015; Richardson et al., 2018; Sharma, 2022; Suksomboon et al., 2019; Yang et al., 2020; Zuraini et al., 2021).

A systematic review and meta‐analytical approach offers a robust approach to consolidate the existing literature, providing a quantitative assessment of the effects of kiwifruit consumption on key cardiometabolic parameters, including body weight, BMI, lipid profiles, blood pressure, and glycemic control. Accordingly, this systematic review and meta‐analysis sought to critically evaluate the current body of evidence regarding the effects of kiwifruit consumption on anthropometric and cardiometabolic indices in adults. By synthesizing data from relevant studies, we aimed to elucidate the potential impact of kiwifruit intake on key health outcomes, thus contributing to a better understanding of its role in cardiometabolic health. The findings of this comprehensive analysis may have implications for dietary recommendations and preventive strategies aimed at reducing the risk of cardiometabolic diseases in the adult population.

## 2 MATERIALS AND METHODS

### 2.1 Literature search

The present study was written according to Preferred Reporting Items for Systematic reviews and Meta‐Analyses (PRISMA) protocols (Page et al., 2021).

### 2.2 Search strategy

A comprehensive online search was conducted on the medical databases, including PubMed, ISI Web of Science, and Scopus, to detect relevant Randomized Clinical Trials (RCTs) and novel systematic reviews of kiwi in adults up to August 2023. The keywords kiwifruit OR “actinidia chinensis” OR “actinidia deliciosa” OR “actinidia kolomikta” OR “green kiwifruit” OR “gold kiwifruit” OR “chinese gooseberry” AND Intervention OR “Intervention Study” OR “Intervention Studies” OR “controlled trial” OR randomized OR random OR randomly OR placebo OR “clinical trial” OR Trial OR “randomized controlled trial” OR “randomized clinical trial” OR RCT OR blinded OR “double blind” OR “double blinded” OR trial OR “clinical trial” OR trials OR “Pragmatic Clinical Trial” OR “Cross‐Over Studies” OR “Cross‐Over Study” OR “Cross‐Over” OR parallel OR “parallel study” OR “parallel trial” were used. No restrictions were applied to the searches of different databases in terms of date and language. A manual search of references from review articles was performed to find missing studies.

### 2.3 Study selection

The study selection process was independently conducted in two phases by two authors (N.H. and O.A.). The initial phase involved screening articles based on their titles and abstracts, and excluding studies that did not meet the eligibility criteria. During the second phase, all the remaining articles were read thoroughly, and only those that met the eligibility criteria were chosen for further review. A third author (MH.H.) contributed to the final decision‐making when there was no agreement on whether to include a study by the two authors. The lists of references in the included articles were analyzed by two authors (N.H. and L.A.).

### 2.4 Eligibility criteria

The inclusion criteria were as follows: (1) adult participants who were >18 years consuming kiwi fruits for ≥2 weeks; (2) inclusion of a control group in which the only difference between the intervention and control groups was the consumption of kiwi fruit; (3) reports of the effects of kiwi fruits consumption on total cholesterol (TC), triglyceride (TG), high‐density lipoprotein cholesterol (HDL‐C), low‐density lipoprotein cholesterol (LDL‐C), fasting blood glucose (FBG), C‐reactive protein (CRP), body weight (BW), body mass index (BMI), and waist circumference (WC) as the primary or secondary outcomes; (4) having an RCT design; and (5) no inclusion of nutraceuticals made from kiwifruit, focusing solely on the direct consumption of whole kiwifruit or kiwifruit juice, and excluding any combination of kiwifruit with other substances or fruits as part of a multicomponent intervention in any of the trials or controls.

### 2.5 Risk of bias

The Cochrane risk of bias checklist for RCTs was used to assess the quality of eligible studies. Two independent authors, O.A. and M.H.H., utilized the following checklist to categorize each included article into one group (low, moderate, or high risk of bias); this checklist actually evaluates the following six sources of bias: random sequence generation, allocation concealment, performance bias, attrition bias, reporting bias, and other causes of bias.

### 2.6 Data extraction

The following data were extracted from included articles by two authors (N.H. and L.A.) independently: first author, year of publication, country, study design, participant, sex, sample size in each group, trial duration, mean age, intervention type and dose, as well as the mean and standard deviation (SD) of TC, TG, HDL‐C, LDL‐C, FBG, CRP, BW, BMI, and WC concentrations in the pre‐ and post‐intervention phases.

### 2.7 Statistical analysis

In this meta‐analysis, we performed statistical analyses using STATA statistical software (version 14; STATA Corp LP). For ascertaining the total effect sizes, weighted mean differences (WMD) and the SD of measures from trial and control groups were extracted using the random effects model according to the DerSimonian and Laird method (DerSimonian & Laird, 2015). We analyzed the differences in study parameters between the intervention and control groups from the beginning to the end of the trial process. Pre‐specified subgroup analyses were performed according to baseline TC, TG, HDL‐C, LDL‐C, FBG, CRP, BW, BMI, and WC, trial duration (≥8 vs. <8 weeks), dose of kiwi, and also the sex of the participants (both male). Sensitivity analyses were conducted in order to evaluate the consistency of the results by excluding one study at a time and determining the influence of each individual article on the overall effect size. The identification of publication bias was carried out using funnel plots and Egger's regression test. A *p*‐value of <.05 was, a priori, considered statistically significant.

### 2.8 Certainty assessment

Grading of Recommendations Assessment, Development, and Evaluation (GRADE) (Guyatt et al., 2008) is used to check the quality of five domains, that is, Risk of bias, Inconsistency, Indirectness, Imprecision, and Publication bias, and finally, the quality of evidence is graded as high, moderate, low, or very low.

## 3 RESULTS

### 3.1 Study selection

**FIGURE 1:**
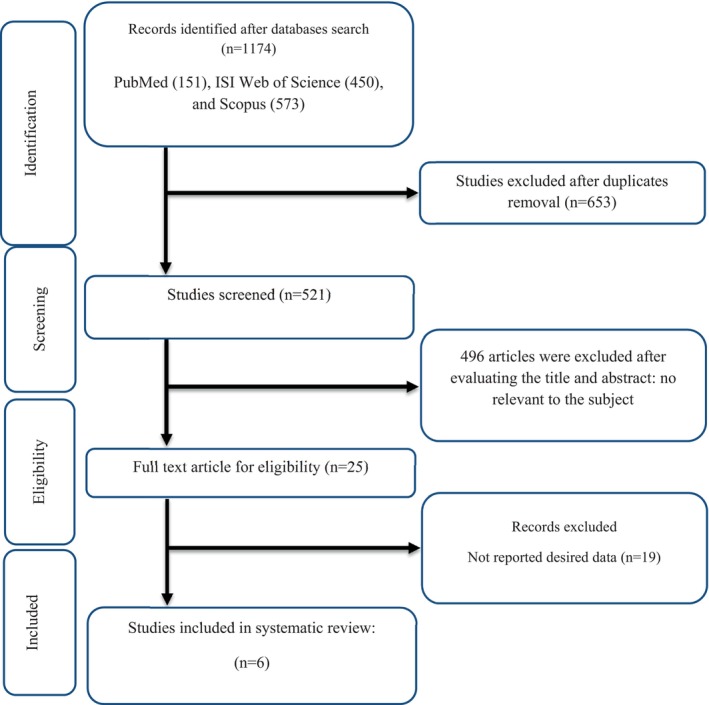
Flow chart of study selection for inclusion trials in the systematic review.

In the initial search, we found a total of 1174 publications in Scopus (573), PubMed (151), and ISI Web of Science (450). Of these, 653 articles were found to be duplicates. Therefore, a total of 521 articles underwent evaluation for the screening of their title and abstract. Following the assessment of the title and abstract, a total of 496 studies that were unrelated were eliminated based on the initial evaluation of the criteria for inclusion. Due to this, a total of 25 studies were obtained and reviewed in full text, of which 19 were excluded because they did not contain the required data. Therefore, a total of 6 RCTs were found to be suitable for inclusion in the present systematic review and meta‐analysis. The flow chart of study selection for inclusion trials in the systematic review is shown in Figure 1.

### 3.2 Study characteristics

Six RCTs were found that evaluated the impact of kiwifruits on metabolic profiles and anthropometric indices. Studies included in the analysis were carried out in different countries, including New Zealand (*n* = 4) (Gammon et al., 2013, 2014; Mishra, Bentley‐Hewitt, et al., 2022; Monro et al., 2022), China (*n* = 1) (Sun et al., 2017), and Italy (*n* = 1) (Graziani et al., 2018). All of the studies had a parallel design, except for two of them, which had a crossover design (Gammon et al., 2013, 2014). Publication dates ranged from 2013 to 2022. The durations of the follow‐up periods varied between 7 weeks and 36 weeks, and the included studies had sample sizes ranging from 32 to 107. Two studies included only male participants (Gammon et al., 2013, 2014), while the remaining articles involved both sexes. The two types of intervention administered were kiwifruit juice (Sun et al., 2017) and green kiwifruits per day, along with a healthy diet. Two studies were conducted on hypercholesterolemic men (Gammon et al., 2013, 2014), one study on subjects with periodontitis (Graziani et al., 2018), two studies on subjects with Type 2 Diabetes Mellitus (Sun et al., 2017) and pre‐diabetes (Mishra, Bentley‐Hewitt, et al., 2022), and one study on healthy people (Monro et al., 2022). The characteristics of the included studies are summarized in Table 1, while the results of the quality assessment are displayed in Table 2.

**TABLE 1:** Characteristic of included studies in meta‐analysis.

Studies	Country	Study design	Participant	Sex	Sample size		Trial duration (week)	Means age IG		Means BMI		Intervention		
					IG	CG		IG	CG	IG	CG	Type	Dose	Control group
Gammon et al. (2013)	New Zealand	Cross‐over, R, DB	Hypercholesterolaemic men	M	44	43	8	48 ± 6.7	48 ± 6.6	27.4 ± 2.87	27.3 ± 2.67	Green kiwifruits per day plus healthy diet	2	Healthy diet
Gammon et al. (2014)	New Zealand	Cross‐over, R, DB	Hypercholesterolaemic men with low inflammation	M	38	22	8	47.7 ± 17.61	48 ± 6.6	25.9 ± 2.51	27.3 ± 2.67	Green kiwifruits per day plus healthy diet	2	Healthy diet
Gammon et al. (2014)	New Zealand	Cross‐over, R, DB	Hypercholesterolaemic men with medium inflammation	M	32	21	8	47.6 ± 9.52	48 ± 6.6	28.5 ± 3.89	27.3 ± 2.67	Green kiwifruits per day plus healthy diet	2	Healthy diet
Sun et al. (2017)	China	Parallel, R, PC	Type 2 Diabetes Mellitus	M/F(M:76,F:31)	55	52	36	56.1 ± 14.4	57.5 ± 12.3	29.3 ± 3.6	30.1 ± 2.9	Kiwifruits juice	10 mL	Placebo
Sun et al. (2017)	China	Parallel, R, PC	Type 2 Diabetes Mellitus	M/F(M:76,F:31)	55	52	24	56.1 ± 14.4	57.5 ± 12.3	29.3 ± 3.6	30.1 ± 2.9	Kiwifruits juice	10 mL	Placebo
Sun et al. (2017)	China	Parallel, R, PC	Type 2 Diabetes Mellitus	M/F(M:76,F:31)	55	52	12	56.1 ± 14.4	57.5 ± 12.3	29.3 ± 3.6	30.1 ± 2.9	Kiwifruits juice	10 mL	Placebo
Graziani et al. (2018)	Italy	Parallel, R,SB	Subjects with periodontitis	M/F(M:19,F:31)	25	25	20	52.4 ± 9.2	50.4 ± 12.7	23.9 ± 4.4	24.4 ± 3.6	Kiwifruits	2	Control
Graziani et al. (2018)	Italy	Parallel, R,SB	Subjects with periodontitis	M/F(M:19,F:31)	25	25	8	52.4 ± 9.2	50.4 ± 12.7	23.9 ± 4.4	24.4 ± 3.6	Kiwifruits	2	Control
Monro et al. (2022)	New Zealand	Parallel, R, PC, DB	Healthy	M/F(M:11,F:32)	20	22	7	21.9 ± 3.5	21.9 ± 2	22.4 ± 2.5	21.6 ± 3.4	Kiwifruits	2	Carbonated water
Mishra et al. (2022)	New Zealand	Parallel, R, PC, SB	Pre‐Diabetes	M/F(M:14,F:18)	17	15	12	55.3 ± 8.3	57 ± 10.9	30.5 ± 7.2	30.6 ± 5.7	Kiwifruits	2	Water

Abbreviations: CG, control group; CO, controlled; DB, double‐blinded; F, Female; IG, intervention group; M, Male; NR, not reported; PC, placebo‐controlled; RA, randomized; SB, single‐blinded.

**TABLE 2:** Risk of bias assessment.

Study	Random sequence generation	Allocation concealment	Selective reporting	Other sources of bias	Blinding (participants and personnel)	Blinding (outcome assessment)	Incomplete outcome data	General risk of bias
Gammon et al. (2013)	L	L	L	L	L	U	L	L
Gammon et al. (2014)	L	L	H	L	L	U	H	L
Gammon et al. (2014)	L	L	H	L	L	U	H	L
Sun et al. (2017)	U	L	H	L	H	U	L	U
Sun et al. (2017)	U	L	H	L	H	U	L	U
Sun et al. (2017)	U	L	H	L	H	U	L	U
Graziani et al. (2018)	L	L	H	H	H	U	L	L
Graziani et al. (2018)	L	L	H	H	H	U	L	L
Monro et al. (2022)	L	L	L	L	L	U	L	L
Mishra et al. (2022)	L	L	L	L	H	U	L	L

*Note*: General Low risk <2 high risk. General moderate risk = high risk. General high risk >2 high risk.

Abbreviations: L; low risk of bias; H, high risk of bias; U, unclear risk of bias.

### 3.3 Effect of kiwi fruit intake on cardiometabolic indices

#### 3.3.1 TG

**FIGURE 2:**
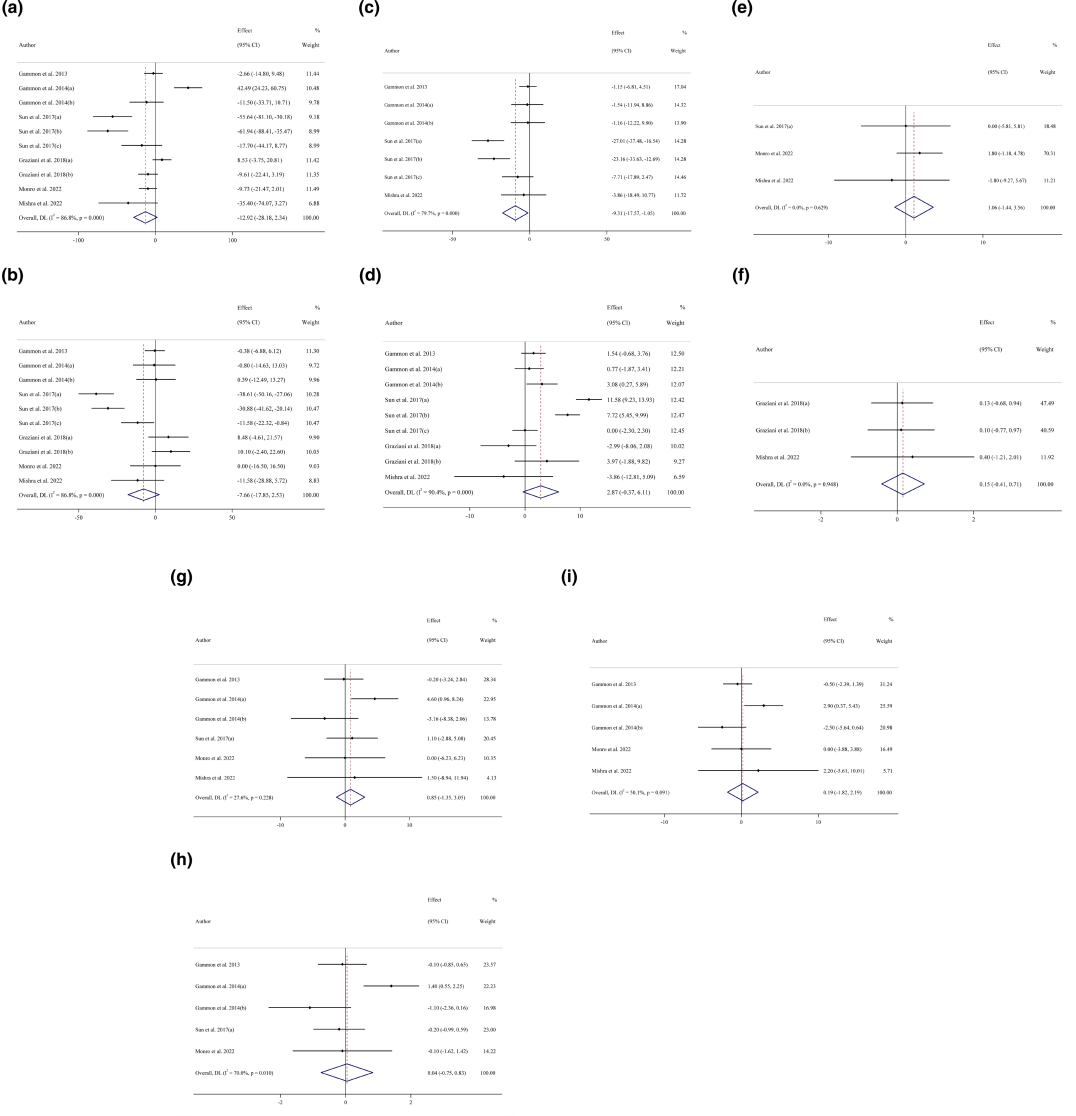
Forest plot detailing weighted mean difference and 95% confidence intervals (CIs) for the effect of kiwi fruit intake on (a) TG (mg/dL); (b) TC (mg/dL); (c) LDL (mg/dL); (d) HDL (mg/dL); (e) FBG (mg/dL); (f) CRP (mg/L); (g) Body weight (kg); (h) BMI (kg/m^2^); and (i) WC (cm).

**FIGURE 3:**
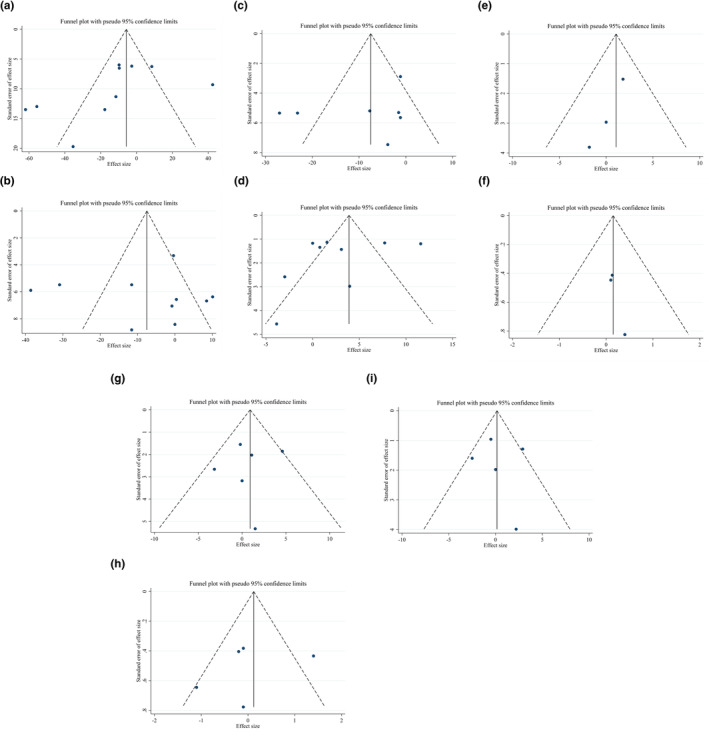
Funnel plots for the effect of kiwi fruit intake on (a) TG (mg/dL); (b) TC (mg/dL); (c) LDL (mg/dL); (d) HDL (mg/dL); (e) FBG (mg/dL); (f) CRP (mg/L); (g) Body weight (kg); (h) BMI (kg/m^2^); and (i) WC (cm).

Overall, 10 effect sizes were assessed to determine the impact of consuming kiwi fruit on TG levels. Pooled effect sizes from the random‐effects model revealed no significant effect of kiwi fruit intake on TG (WMD: −12.91 mg/dL; 95% CI: (−28.17 to 2.34, *p* = .097)) compared with the control group, with significant heterogeneity between studies (*I* ^2^ = 86.8%, *p* < .001) (Figures 2 and 3).

However, after subgroup analysis based on TG level, we observed a significant effect of kiwi fruit intake on TG group ≥150 (WMD: −36.06 mg/dL; 95% CI: (−57.44 to −14.67, *p* = .001)), with moderate heterogeneity (*I* ^2^ = 67.8%, *p* = .014). In addition, subgroup analysis based on sex demonstrated that kiwi fruit intake had a significantly decreasing effect on TG in both sexes (WMD: −23.00 mg/dL; 95% CI: (−40.38 to −5.63, *p* = .001), *I* ^2^ = 84.0%, *p* heterogeneity <.001) and no effect on males only (Table 3).

**TABLE 3:** Subgroup analyses of kiwi fruit intake on metabolic profile and anthropometric indices in adults.

	Number of effect sizes	WMD (95%CI)	*p*‐value	Heterogeneity		
				*p* heterogeneity	*I* ^2^	*p* between sub‐groups
*Kiwi fruit intake on TG level* (*mg/dL*)						
Overall effect	10	−12.91 (−28.17, 2.34)	.097	<.001	86.8%	
TG						
<150	5	4.76 (−10.62, 20.15)	.544	<.001	85.4%	.002
>150	5	−36.06 (−57.44, −14.67)	**.001**	.014	67.8%	
Trial duration (week)						
≤8	5	1.32 (−15.48, 18.13)	.877	<.001	84.9%	.078
>8	5	−31.42 (−63.68, 0.83)	.056	<0.001	89.2%	
Baseline BMI (kg/m^2^)						
Normal (18.5–24.9)	3	−3.60 (−15.50, 8.28)	.552	.060	64.5%	.299
Overweight (25–29.9)	7	−19.04 (−45.60, 7.52)	.160	<.001	90.3%	
Sex						
Both	7	−23.00 (−40.38, −5.63)	**.009**	<.001	84.0%	.074
Male	3	9.53 (−21.58, 40.64)	.548	<.001	89.8%	
*Kiwi fruit intake on TC level* (*mg/dL*)						
Overall effect	10	−7.66 (−17.85, 2.52)	.141	<.001	86.8%	
TC						
<200	1	0.00 (−16.50, 16.50)	1.000	–	–	.327
>200	7	−10.68 (−24.24, 2.87)	.123	<.001	90.6%	
Trial duration (week)						
≤8	5	1.19 (−3.51, 5.91)	.619	.682	0.0%	.035
>8	5	−17.12 (−33.48, −0.77)	**.040**	<.001	88.7%	
Baselin BMI (kg/m^2^)						
Normal (18.5–24.9)	3	7.17 (−0.75, 15.10)	.076	.614	0.0%	.006
Overweight (25–29.9)	7	−13.40 (−25.59, −1.21)	**.031**	<.001	88.1%	
Sex						
Both	7	−10.85 (−25.54, 3.82)	.147	<.001	89.2%	.186
Male	3	−0.31 (−5.66, 5.04)	.910	.992	0.0%	
*Kiwi fruit intake on LDL level* (*mg/dL*)						
Overall effect	7	−9.30 (−17.56, −1.04)	**.027**	<.001	79.7%	
LDL						
<130	1	−3.86 (−18.48, 10.76)	.605	–	–	.295
>130	4	−14.33 (−27.39, −1.27)	**.041**	<.001	84.8%	
Trial duration (week)						
≤8	3	−1.22 (−5.75, 3.30)	.596	.998	0.0%	.014
>8	4	−15.97 (−26.82, −5.13)	**.004**	.011	73.1%	
Sex						
Both	4	−15.97 (−26.82, −5.13)	**.004**	.011	73.1%	.014
Male	3	−1.22 (−5.75, 3.30)	.596	.998	0.0%	
*Kiwi fruit intake on HDL level* (*mg/dL*)						
Overall effect	9	2.87 (−0.36, 6.11)	0.082	<.001	90.4%	
Trial duration (week)						
≤8	4	1.84 (0.43, 3.26)	**.010**	.581	0.0%	.674
>8	5	3.10 (−2.58, 8.79)	.285	<.001	94.0%	
Baselin BMI (kg/m^2^)						
Normal (18.5–24.9)	2	0.33 (−6.48, 7.14)	.924	.078	67.8%	.425
Overweight (25–29.9)	7	3.47 (−0.15, 7.09)	.060	<.001	92.1%	
Sex						
Both	6	3.27 (−1.75, 8.30)	.201	<.001	92.5%	.559
Male	3	1.71 (0.26, 3.17)	.021	.491	0.0%	
*Kiwi fruit intake on FBG level* (*mg/dL*)						
Overall effect	3	1.06 (−1.43, 3.56)	.404	.629	0.0%	
*Kiwi fruit intake on CRP level* (*mg/L*)						
Overall effect	3	0.15 (−0.40, 0.70)	.0598	.948	0.0%	
*Kiwi fruit intake on body weight* (*kg*)						
Overall effect	6	0.85 (−1.34, 3.04)	.448	.228	27.6%	
Trial duration (week)						
≤8	4	0.61 (−2.64, 3.86)	.714	.076	56.4%	.830
>8	2	1.15 (−2.56, 4.86)	.544	.944	0.0%	
Baselin BMI (kg/m^2^)						
Normal (18.5–24.9)	1	0.00 (−6.23, 6.23)	1.000	–	–	.790
Overweight (25–29.9)	5	0.91 (−1.66, 3.49)	.486	.146	41.3%	
Sex						
Both	3	0.84 (−2.34, 4.04)	.602	.950	0.0%	.947
Male	3	0.67 (−3.44, 4.79)	.749	.033	70.6%	
*Kiwi fruit intake on BMI* (*kg/m* ^2^)						
Overall effect	5	0.04 (−0.75, 0.83)	.920	.010	70.0%	
BMI						
1	1	−0.10 (−1.62, 1.42)	.898	–	–	.864
2	4	0.05 (−0.87, 0.98)	.906	.004	77.3%	
Trial duration (week)						
≤8	4	0.09 (−0.96, 1.14)	.868	.006	75.9%	.667
>8	1	−0.20 (−0.99, 0.59)	.621	–	–	
Baselin BMI (kg/m^2^)						
Normal (18.5–24.9)	1	−0.10 (−1.62, 1.42)	.898	–	–	.864
Overweight (25–29.9)	4	0.05 (−0.87, 0.98)	.906	.004	77.3%	
Sex						
Both	2	−0.17 (−0.88, 0.52)	.618	.909	0.0%	.693
Male	3	0.12 (−1.20, 1.45)	.855	.002	83.6%	
*Kiwi fruit intake on WC* (*cm*)						
Overall effect	5	0.18 (−1.81, 2.19)	.855	.091	50.1%	
Trial duration (week)						
≤8	4	0.05 (−2.14, 2.25)	.960	.052	61.2%	.605
>8	1	2.20 (−5.61, 10.01)	.581	–	–	
Baselin BMI (kg/m^2^)						
Normal (18.5–24.9)	1	0.00 (−3.88, 3.88)	1.000	–	–	.920
Overweight (25–29.9)	4	0.23 (−2.27, 2.74)	.853	.046	62.5%	
Sex						
Both	2	0.43 (−3.04, 3.91)	.806	.621	0.0%	.866
Male	3	0.04 (−2.76, 2.86)	.973	.021	74.2%	

*Note:* The significant p‐values made bold.

Abbreviations: CI, confidence interval; WMD, weighted mean differences.

#### 3.3.2 TC

Overall, 10 effect sizes were assessed to determine the impact of consuming kiwi fruit on TC levels. Pooled effect size from the random‐effects model revealed no significant effect of kiwi fruit intake on TC (WMD: −7.66 mg/dL; 95% CI: (−17.85 to 2.52, *p* = .141)) compared with the control group, with significant heterogeneity between studies (*I* ^2^ = 86.8%, *p* < .001). However, subgroup analysis revealed that trial durations of more than 8 weeks had a significantly decreasing effect on TC (WMD: −17.12 mg/dL; 95% CI: (−33.48 to −0.77, *p* = .040), *I* ^2^ = 88.7%, *p* heterogeneity <.001). In addition, subgroup analysis conducted according to participants' baseline BMI revealed that kiwi fruit intake had a significant effect on overweight (25–29.9) subjects (WMD: −13.40 mg/dL; 95% CI: (−25.59 to −1.21, *p* = .031), *I* ^2^ = 88.1%, *p* heterogeneity <.001) (Table 3).

#### 3.3.3 LDL

Overall, 7 effect sizes were assessed to determine the impact of consuming kiwi fruit on LDL levels. Pooled effect sizes from the random‐effects model revealed a significant decreasing effect of kiwi fruit intake on LDL (WMD: −9.30 mg/dL; 95% CI: (−17.56 to −1.04, *p* = .027)) compared with the control group, with significant heterogeneity between studies (*I* ^2^ = 79.7%, *p* < .001). Moreover, subgroup analysis showed that kiwi fruit intake reduced LDL in all subgroups (Table 3).

#### 3.3.4 HDL

Overall, 9 effect sizes were assessed to determine the impact of consuming kiwi fruit on HDL levels. Pooled effect sizes from the random‐effects model revealed no significant effect of kiwi fruit intake on HDL (WMD: 2.87 mg/dL; 95% CI: −0.36 to 6.11, *p* = .141) compared with the control group, with significant heterogeneity between studies (*I* ^2^ = 90.4%, *p* < .001). However, subgroup analysis revealed that trial durations ≤8 weeks had a significant effect on HDL (WMD: 1.84 mg/dL; 95% CI: 0.43 to 3.26, *p* = .010, *I* ^2^ = 0.0%, *p* heterogeneity = 0.581) (Table 3).

#### 3.3.5 FBG

Overall, 3 effect sizes were assessed to determine the impact of consuming kiwi fruit on FBG. Pooled effect sizes from the random‐effects model revealed no significant effect of kiwi fruit intake on FBG (WMD: 1.06 mg/dL; 95% CI: −1.43 to 3.56, *p* = .404) compared with the control group, with no significant heterogeneity between studies (*I* ^2^ = 0.0%, *p* = .629).

#### 3.3.6 CRP

Overall, 3 effect sizes were assessed to determine the impact of consuming kiwi fruit on CRP. Pooled effect sizes from the random‐effects model revealed no significant effect of kiwi fruit intake on CRP (WMD: 0.15 mg/dL; 95% CI: −0.40, 0.70, *p* = .0598) compared with the control group, with no significant heterogeneity between studies (*I* ^2^ = 0.0%, *p* = .948).

### 3.4 Effect of kiwi fruit intake on anthropometric indices

#### 3.4.1 Body weight

Overall, 6 effect sizes were assessed to determine the impact of consuming kiwi fruit on body weight. Pooled effect sizes from the random‐effects model revealed no significant effect of kiwi fruit intake on body weight (WMD: 0.85 kg; 95% CI: (−1.34 to 3.04, *p* = .448)) compared with the control group. There was no significant heterogeneity between studies (*I* ^2^ = 27.6%, *p* = .228) (Table 3).

#### 3.4.2 BMI

Overall, 5 effect sizes were assessed to determine the impact of consuming kiwi fruit on BMI. Pooled effect sizes from the random‐effects model revealed no significant effect of kiwi fruit intake on BMI (WMD: 0.04 kg/m^2^; 95% CI: −0.75 to 0.83, *p* = .920) compared with the control group, with significant heterogeneity between studies (*I* ^2^ = 70.0%, *p* = .010) (Table 3).

#### 3.4.3 WC

Overall, 5 effect sizes were assessed to determine the impact of consuming kiwi fruit on WC. Pooled effect sizes from the random‐effects model revealed no significant effect of kiwi fruit intake on WC (WMD: 0.18 cm; 95% CI: −1.81 to 2.19, *p* = .855) compared with the control group, with moderate heterogeneity between studies (*I* ^2^ = 50.1%, *p* = .091) (Table 3).

#### 3.4.4 Publication bias and sensitivity analyses

According to Egger's regression test, it was indicated that there was publication bias for TG and WC (Table 4). However, the sensitivity analyses revealed that none of the individual studies had a significant impact on the overall effect sizes of TG and WC.

**TABLE 4:** GRADE profile of kiwi fruit intake on metabolic profile and anthropometric indices in adults.

Outcomes	Risk of bias	Inconsistency	Indirectness	Imprecision	Publication bias	Quality of evidence
TG	No serious limitation	Very serious limitation^1^	No serious limitation	Serious limitation^1^	No serious limitation	⊕◯◯◯ Low
TC	No serious limitation	Very serious limitation^1^	No serious limitation	Serious limitation^1^	No serious limitation	⊕◯◯◯ Low
LDL	No serious limitation	Very serious limitation^1^	No serious limitation	No serious limitation	No serious limitation	⊕⊕◯◯ Moderate
HDL	No serious limitation	Very serious limitation^1^	No serious limitation	Serious limitation^1^	No serious limitation	⊕◯◯◯ Low
FPG	No serious limitation	No serious limitation	No serious limitation	Serious limitation^1^	No serious limitation	⊕⊕⊕◯ High
CRP	No serious limitation	No serious limitation	No serious limitation	Serious limitation^1^	No serious limitation	⊕⊕⊕◯ High
Body weight	No serious limitation	No serious limitation	No serious limitation	Serious limitation^1^	No serious limitation	⊕⊕⊕◯ High
BMI	No serious limitation	Serious limitation^1^	No serious limitation	Serious limitation^1^	No serious limitation	⊕⊕◯◯ Moderate
WC	No serious limitation	Serious limitation^1^	No serious limitation	Serious limitation^1^	No serious limitation	⊕⊕◯◯ Moderate

^a^ There is high heterogeneity (*I* ^2^ > 40%).

^b^ There is high heterogeneity (*I* ^2^ > 75%).

^c^ There is no evidence of significant effects of kiwi fruit intake.

^d^ There is a significant publication bias based on Egger's test.

#### 3.4.5 Grading of evidence

The certainty of the evidence was determined using the GRADE protocol (Table 4). The evaluation of inconsistency and publication bias led to a score of low quality for all factors due to severe heterogeneity.

## 4 DISCUSSION

Kiwifruit is popular for being a nutritious source that is high in fiber, potassium, vitamin C, and various phytochemicals, like carotenoids (lutein and ß‐carotene), flavonoids, anthocyanins, and tocols (α‐tocopherol and γ‐tocotrienol) (D'Evoli et al., 2015; Satpal et al., 2021; Svendsen et al., 2015). Extensive research studies have revealed that kiwifruit is abundant in nutrients that offer numerous health benefits to those who consume it, enhancing an individual's digestive, immune, and metabolic health (Richardson et al., 2018). Phytochemicals have the ability to influence various processes that help protect against oxidative stress and DNA damage. These processes include cell signaling, gene expression, and enzyme activity. Kiwifruit may also aid in protecting against arteriosclerosis, a multifaceted condition that includes cholesterol oxidation, intracellular accumulation of oxidized cholesterol, elevated blood pressure, and aggregation of platelets (Hunter et al., 2016).

Several studies have also examined the impact of consuming kiwifruit on metabolic abnormalities, including dyslipidemia, which is characterized by elevated levels of total cholesterol (TC), low‐density lipoprotein cholesterol (LDL‐C), triglycerides (TG), and decreased levels of high‐density lipoprotein cholesterol (HDL‐C) (Alim et al., 2020). LDL‐C has been recognized as a significant contributor to cardiovascular disease in numerous epidemiological and interventional investigations, given its crucial role in the development of atherosclerosis. In recent times, LDL‐C has largely supplanted TC as the principal lipid measurement for assessing cardiovascular risk (Jung et al., 2022).

For example, Chang and Liu (2009) conducted a clinical‐based study in Taiwan to examine how consuming two kiwifruits (100 g each) affected the lipid profile, antioxidants, and markers of lipid peroxidation in adults with hyperlipidemia. After 8 weeks of the intervention, there was a significant increase in HDL‐C concentration, concomitant with significant decreases in both the LDL‐C/HDL‐C ratio and the TC/HDL‐C ratio. Also, the levels of vitamin C and vitamin E, which are antioxidant nutrients, as well as the overall antioxidant status in the plasma, also showed a significant increase in fasting blood samples.

A previous study was conducted using a randomized crossover design to evaluate the effects of kiwifruit on platelet activity and lipid profile in healthy individuals. Accordingly, the authors reported that the consumption of kiwi fruit reduced blood triglyceride levels by 15% in comparison to the control group, while no similar effects were seen with regard to cholesterol levels (Duttaroy & Jørgensen, 2004). Additionally, in a study conducted by Yang et al. (2020), it was demonstrated that the regular intake of golden kiwifruit on a daily basis for a period of 6 weeks can lead to a decrease in body fat mass and blood pressure, as well as the control and management of inflammatory reactions among young adults who are overweight or obese. Furthermore, in a study conducted by Gammon et al. (2013), it was reported that consuming two green kiwifruits daily for a duration of 4 weeks resulted in positive impacts on plasma HDL‐C levels and the TC:HDL‐C ratio, as compared to a healthy control diet.

The improvements in dyslipidemia may be due to the independent and/or synergistic effects of various components present in kiwifruit, including polyphenols, vitamin C, and vitamin E (Stonehouse et al., 2012). Additionally, research has shown that polyphenols derived from fruits and vegetables can effectively reduce LDL levels and oxidative stress and raise HDL concentrations (Alim et al., 2020). In addition, kiwifruit contains not only polyphenols but also high amounts of dietary fiber, which has been shown in previous studies to help reduce lipid and cholesterol levels (He et al., 2022; Soliman, 2019). However, according to a recent systematic review (Suksomboon et al., 2019), it was found that kiwifruit did not have an impact on metabolic health in individuals with cardiovascular risk factors such as hypercholesterolemia, hypertension, Type 2 diabetes, and smokers. This was determined by measuring SBP, DBP, TC, TG, LDL, HDL, FPG, HOMA‐IR, and body weight.

In the present meta‐analysis, which summarized the findings of six RCTs involving 403 participants, we found that there was no significant reduction in body weight, BMI, and WC among participants who consumed kiwifruit. Additionally, the subgroup analysis did not reveal any significant changes in the results.

Furthermore, we found that the consumption of kiwifruits leads to a significant reduction in LDL cholesterol levels when compared to baseline levels. Nevertheless, there were no significant differences observed in TG, total cholesterol (TC), HDL cholesterol, FBG, and CRP between the baseline and final assessments. However, the results did significantly change when we conducted subgroup analysis according to TG levels, sex, trial duration, and BMI. For instance, in a sub‐group analysis based on baseline BMI, it was observed that consuming kiwi fruit had a significant impact on reducing TC levels among overweight (BMI = 25–29.9) participants.

The present study included a diverse range of participants, such as individuals with Type 2 Diabetes Mellitus in one study and hypercholesterolemic men in another study. It should be noted that two types of kiwifruit were used, namely whole fruits (2 per day) and kiwifruit juice (100 mL per day).

There are a few limitations that need to be addressed in the current study. Firstly, our analysis shows statistical heterogeneity. Secondly, the majority of the studies did not assess or regulate the participants' dietary intake and levels of physical activity. Consequently, interpretation of the findings and determining the specific impact of kiwifruit on these factors alone were challenging. Additionally, the included studies were restricted to participants from New Zealand, China, or Italy, which limits generalizability.

## 5 CONCLUSION

In conclusion, the current study does not show a significant impact of kiwifruit consumption on reducing weight or WC. We also discovered that the consumption of kiwifruit did not significantly impact cardiometabolic indices, with the exception of LDL‐C levels. However, it is important to approach the findings cautiously due to the limitations of the existing studies and the high heterogeneity in the results of the study.

## AUTHOR CONTRIBUTIONS

**Pedram Pam:** Data curation (equal); investigation (equal); writing – original draft (equal). **Mohammad Ali Goudarzi:** Data curation (equal); resources (equal). **Shirin Ghotboddin Mohammadi:** Validation (equal); visualization (equal). **Omid Asbaghi:** Formal analysis (equal); software (equal). **Ladan Aghakhani:** Investigation (equal); writing – original draft (equal). **Cain C. T. Clark:** Writing – review and editing (equal). **Mohammad Hashem Hashempur:** Conceptualization (equal); project administration (equal); writing − original draft; supervision (equal). **Neda Haghighat:** Methodology (equal); project administration (equal); writing – original draft (equal).

## FUNDING INFORMATION

None.

## CONFLICT OF INTEREST STATEMENT

Not applicable.

## Data Availability

The data used to support the findings of this study are available from the corresponding authors upon request.
